# Ring finger protein 2 promotes colorectal cancer progression by suppressing early growth response 1

**DOI:** 10.18632/aging.202396

**Published:** 2020-12-19

**Authors:** Feilong Wei, Haoren Jing, Ming Wei, Lei Liu, Jieheng Wu, Meng Wang, Donghui Han, Fa Yang, Bo Yang, Dian Jiao, Guoxu Zheng, Lingling Zhang, Wenjin Xi, Zhangyan Guo, An-Gang Yang, Weijun Qin, Yi Zhou, Weihong Wen

**Affiliations:** 1 Department of Orthopedics, Tangdu Hospital, Fourth Military Medical University, Xi’an 710038, China; 2 Department of Anorectal Surgery, Tianjin Union Medical Center, Nankai University Affiliated Hospital, Tianjin 300013, China; 3 Urology Department of No. 989 Hospital, Joint Logistics Support Force of PLA, Luoyang 471000, China; 4 Department of Gastroenterology, Tangdu Hospital, Fourth Military Medical University, Xi’an 710038, China; 5 State Key Laboratory of Cancer Biology, Department of Immunology, Fourth Military Medical University, Xi’an 710032, China; 6 Department of Urology, Xijing Hospital, Fourth Military Medical University, Xi’an 710032, China; 7 Department of Urology, Tangdu Hospital, Fourth Military Medical University, Xi’an 710038, China; 8 Institute of Medical Research, Northwestern Polytechnical University, Xi'an 710072, China

**Keywords:** RNF2, EGR1, colorectal cancer, cell proliferation, apoptosis

## Abstract

Ring finger protein 2 (RNF2) is an important component of polycomb repressive complex 1. RNF2 is upregulated in many kinds of tumors, and elevated RNF2 expression is associated with a poor prognosis in certain cancers. To assess the function of RNF2 in colorectal cancer, we examined RNF2 protein levels in 313 paired colorectal cancer tissues and adjacent normal tissues. We then analyzed the association of RNF2 expression with the patients’ clinicopathologic features and prognoses. RNF2 expression was upregulated in colorectal cancer tissues and was associated with the tumor differentiation status, tumor stage and prognosis. In colorectal cancer cell lines, downregulation of RNF2 inhibited cell proliferation and induced apoptosis. Gene microarray analysis revealed that early growth response 1 (EGR1) was upregulated in RNF2-knockdown cells. Knocking down EGR1 partially reversed the inhibition of cell proliferation and the induction of apoptosis in RNF2-knockdown cells. RNF2 was enriched at the EGR1 promoter, where it mono-ubiquitinated histone H2A, thereby inhibiting EGR1 expression. These results indicate that RNF2 is oncogenic in colorectal cancer and may promote disease progression by inhibiting EGR1 expression. RNF2 is thus a potential prognostic marker and therapeutic target in colorectal cancer.

## INTRODUCTION

Colorectal cancer (CRC) is among the most frequently diagnosed cancer types, with a worldwide incidence of >1,000,000 cases per year. Despite the availability of treatments such as surgery, radiotherapy and chemotherapy, CRC is still a major contributor to cancer-associated mortality around the world [1]. Genetic and epigenetic mechanisms may promote the occurrence and progression of CRC [2–4]. Thus, it is critical to further explore the pathways contributing to CRC occurrence and progression so that novel therapeutic targets and strategies can be determined.

Epigenetic regulation is one of the critical mechanisms that induce tumor occurrence and progression [5, 6]. The study of epigenetic regulators can not only reveal their function in tumor occurrence and progression, but also provide novel therapeutic targets. Indeed, as a result of functional studies of epigenetic regulators, the US Food and Drug Administration has approved inhibitors of DNA methyltransferases, histone deacetylases and Janus kinase 2 for particular cancer treatments [7–9].

Polycomb group (PcG) proteins are highly conserved epigenetic modifiers that function in multimeric complexes. In mammals, PcG proteins form two main complexes: polycomb repressive complexes 1 and 2 (PRC1 and PRC2). PRC1 is composed of BMI1, polyhomeotic, polycomb, Ring1a and ring finger protein 2 (RNF2, also known as Ring1b). PRC1 was shown to mono-ubiquitinate K119 of histone H2A [10–12]. PRC2 includes SUZ12, embryonic ectoderm development and enhancer of zeste 2, and was demonstrated to tri-methylate histone H3 at K27 [13, 14]. The modifications induced by PRC1 and PRC2 are transcriptionally repressive, and may influence each other [15]. PcG proteins participate in tumor occurrence and progression [16–18], and those that are oncogenic (such as enhancer of zeste 2 and BMI1) could be used as novel targets for cancer therapy [19–21].

RNF2 has been demonstrated to be the main PRC1 component that promotes H2A mono-ubiquitination at K119 [11]. RNF2 is upregulated in many human cancer types, and elevated RNF2 expression is an independent poor prognostic marker in pancreatic, breast, ovarian and bladder cancers [22–24]. In certain cancer types, RNF2 induces the ubiquitination/destabilization of p53 (directly or through MDM2), and the downregulation of RNF2 can suppress xenograft growth *in vivo* [25, 26]. Several genes have been identified as epigenetic targets of RNF2; for example, cyclin-dependent kinase inhibitors 1A and 2A (CDKN1A and CDKN2A) were shown to be RNF2 targets in hepatic stem/progenitor cells [27], and thioredoxin interacting protein was reported to be an RNF2 target in prostate cancer [28]. However, the involvement of RNF2 in CRC is unclear.

Here, we examined the expression of RNF2 in CRC tumor tissues and evaluated its association with clinicopathologic features and patients’ prognoses. We also studied the function of RNF2 in CRC cell lines by assessing the effects of downregulating RNF2 on cell proliferation and apoptosis. Finally, we explored the oncogenicity of RNF2 in terms of its effects on early growth response 1 (EGR1) expression. Our study indicated that RNF2 could be a novel prognostic marker and therapeutic target in CRC.

## RESULTS

### RNF2 was upregulated in CRC tissues

**Figure 1:**
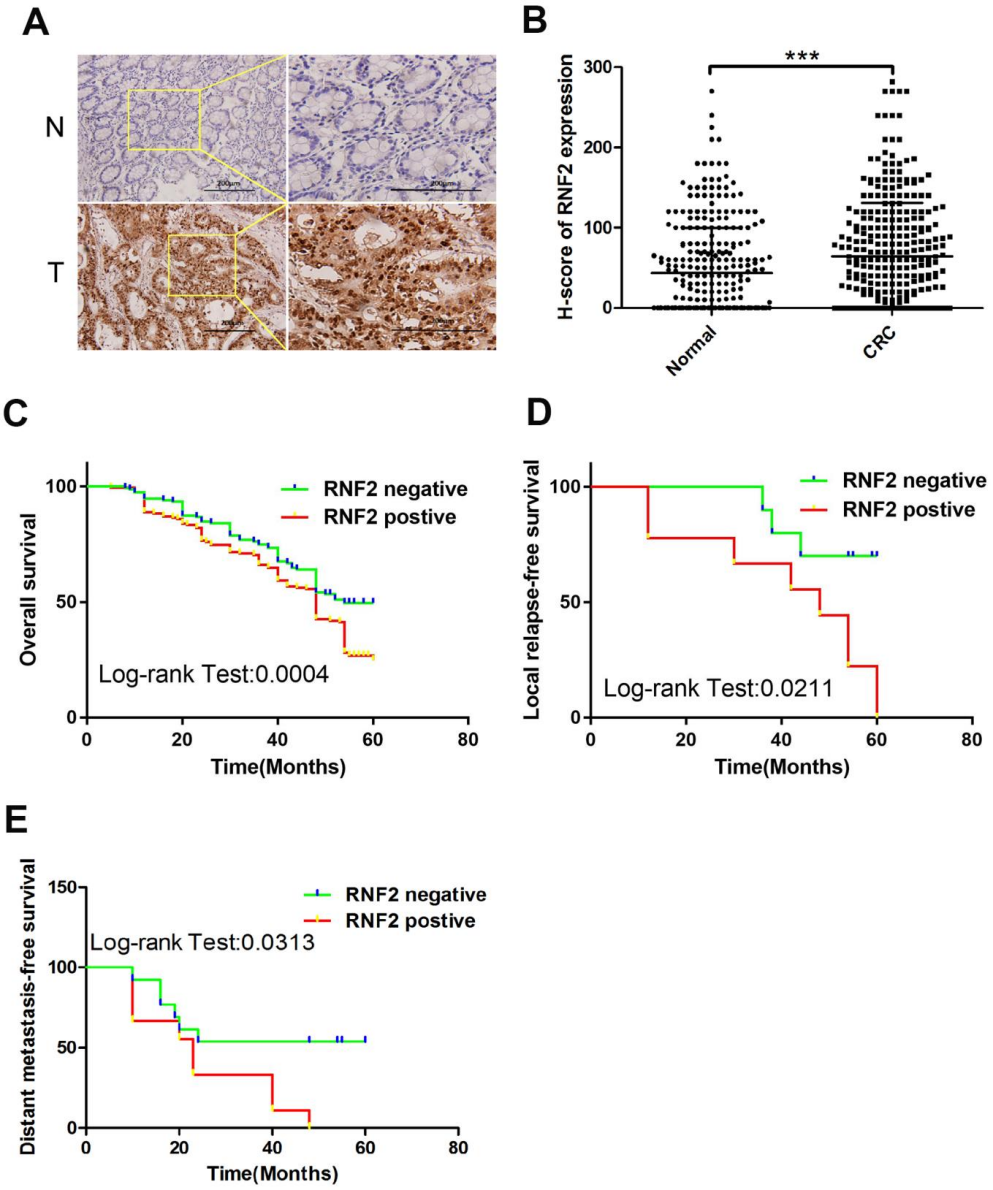
**RNF2 expression was upregulated in CRC tumor tissues and was associated with patients’ prognoses.** (**A**) Representative immunohistochemistry results depicting positive and negative RNF2 staining in clinical CRC tumor tissues and adjacent normal tissues. The pictures on the right are the magnified view of the yellow boxes on the left. Scale bar: 200 μm. (**B**) H-scores for RNF2 expression in 313 CRC tumor tissues and adjacent normal tissues. ***p<0.001 versus adjacent normal tissue. (**C**) Overall survival of RNF2-positive or -negative CRC patients. (**D**) Local relapse-free survival of RNF2-positive or -negative CRC patients. (**E**) Distant metastasis-free survival of RNF2-positive or -negative CRC patients.

Immunohistochemistry was used to examine RNF2 expression in tissue microarrays containing 313 paired CRC tumor tissues and adjacent normal tissues. RNF2 protein levels were noticeably greater in CRC tumor tissues than in adjacent normal tissues (H-scores: 64.40±56.14 and 43.54±66.38, respectively; Figure 1A, 1B). Patients were then separated into two groups based on their RNF2 levels, and were designated as RNF2-positive or RNF2-negative. When we analyzed the association between RNF2 levels and clinicopathologic characteristics, we observed that RNF2 positivity was associated with a significantly worse tumor differentiation status, tumor-node-metastasis (TNM) stage and Duke’s stage (Table 1). These findings demonstrated that RNF2 expression is upregulated and significantly associated with the tumor differentiation status and tumor stage in CRC.

**Table 1:** The correlation between RNF2 expression and clinicopathological features in CRC patients (n=313).

**Clinicopathological features**	**No. of patients (%)**	**RNF2 expression**		**χ2**	***P* value**
		**positive**	**negative**		
**Age (years)**				0.007	0.932
≤ 60	128 (40.9%)	66	62		
> 60	185 (59.1%)	97	88		
**Gender**				0.135	0.713
Male	175 (55.9%)	94	81		
Female	138 (44.1%)	69	69		
**Tumor invasion**				7.933	0.088
T0	58 (18.5%)	48	10		
T1	35 (11.2%)	13	22		
T2	39 (12.5%)	21	18		
T3	176 (56.2%)	79	97		
T4	5 (1.6%)	2	3		
**Lymph node metastasis**				0.906	0.645
N0	226 (72.2%)	120	106		
N1	58 (18.5%)	32	26		
N2	29 (9.3%)	11	18		
**Distant metastasis**				1.183	0.277
M0	291 (93.0%)	154	137		
M1	22 (7.0%)	9	13		
**Tumor differentiation**				24.801	0.001*
Well	52 (16.6%)	21	31		
Moderately	155 (49.5%)	66	89		
Poorly	106 (33.9%)	76	30		
**TNM stage**				5.798	0.016*
I -II	213 (68.1%)	101	112		
III- IV	100 (31.9%)	62	38		
**Dukes stage**				5.632	0.018*
Dukes A-B	198 (63.3%)	93	105		
Dukes C-D	115 (36.7%)	70	45		

*Statistically significant (P < 0.05).

### High RNF2 expression was associated with a poor prognosis in CRC patients

To determine the prognostic value of RNF2 in CRC, we assessed the relationship between RNF2 levels and overall survival. The overall survival time tended to be shorter in RNF2-positive patients than in RNF2-negative patients (Figure 1C). RNF2-positive patients also had shorter five-year local relapse-free survival and distant metastasis-free survival times than RNF2-negative patients (Figure 1D, 1E). In addition, the survival of patients with different tumor differentiation statuses, TNM stages and Duke’s stages differed significantly between the RNF2-positive and RNF2-negative groups (Supplementary Figure 1A–1G).

Next, we performed a Cox regression analysis to determine the prognostic value of RNF2 in CRC patients. As shown in Table 2, in a univariate analysis, RNF2 expression, tumor cell differentiation, the Duke’s stage and the TNM stage were all associated with the CRC prognosis. In a multivariate analysis, RNF2 positivity was associated with reduced overall survival, with an adjusted hazard ratio of 1.621 (95% confidence interval: 1.195-2.198; p=0.002). These results indicated that RNF2 can serve as an independent prognostic marker in CRC.

**Table 2:** Cox regression analysis of prognostic factors for overall survival in CRC patients (n=313).

	**Univariate**				**Multivariate**		
	**HR**	**95% CI**	**P value**		**HR**	**95% CI**	**P value**
**RNF2 expression** (Negative vs. Positive)	1.635	1.225-2.181	0.001*		1.621	1.195-2.198	0.002*
**Age** (> 60 vs. ≤ 60)	1.133	0.849-1.512	0.397		1.119	0.831-1.507	0.459
**Gender** (Female vs. Male)	1.070	0.807-1.419	0.638		0.966	0.721-1.295	0.817
**TNM stage**			0.002*				0.001*
TNM2 vs. TNM1	0.628	0.436-0.905	0.013		0.464	0.305-0.707	0.001
TNM3 vs. TNM1	0.715	0.461-1.109	0.134		0.355	0.203-0.620	0.001
TNM4 vs. TNM1	1.326	0.829-2.121	0.239		0.859	0.509-1.449	0.568
**Differentiation**			0.027*				0.011*
Moderately vs. well	0.771	0.569-1.044	0.092		0.914	0.665-1.257	0.581
Poorly vs. well	0.556	0.356-0.868	0.010		0.485	0.300-0.783	0.003
**Dukes stage**			0.010*				0.031*
Dukes B vs. Dukes A	0.743	0.480-1.153	0.185		0.889	0.545-1.449	0.637
Dukes C vs. Dukes A	1.033	0.660-1.618	0.886		1.365	0.800-2.331	0.254
Dukes D vs. Dukes A	2.037	0.982-4.227	0.056		2.175	0.972-4.866	0.059

Abbreviations: HR, hazard ratio; 95% CI, 95% confidence interval.

*Statistically significant (P < 0.05).

### The downregulation of RNF2 reduced cell proliferation, promoted apoptosis and induced senescence in CRC cells

**Figure 2:**
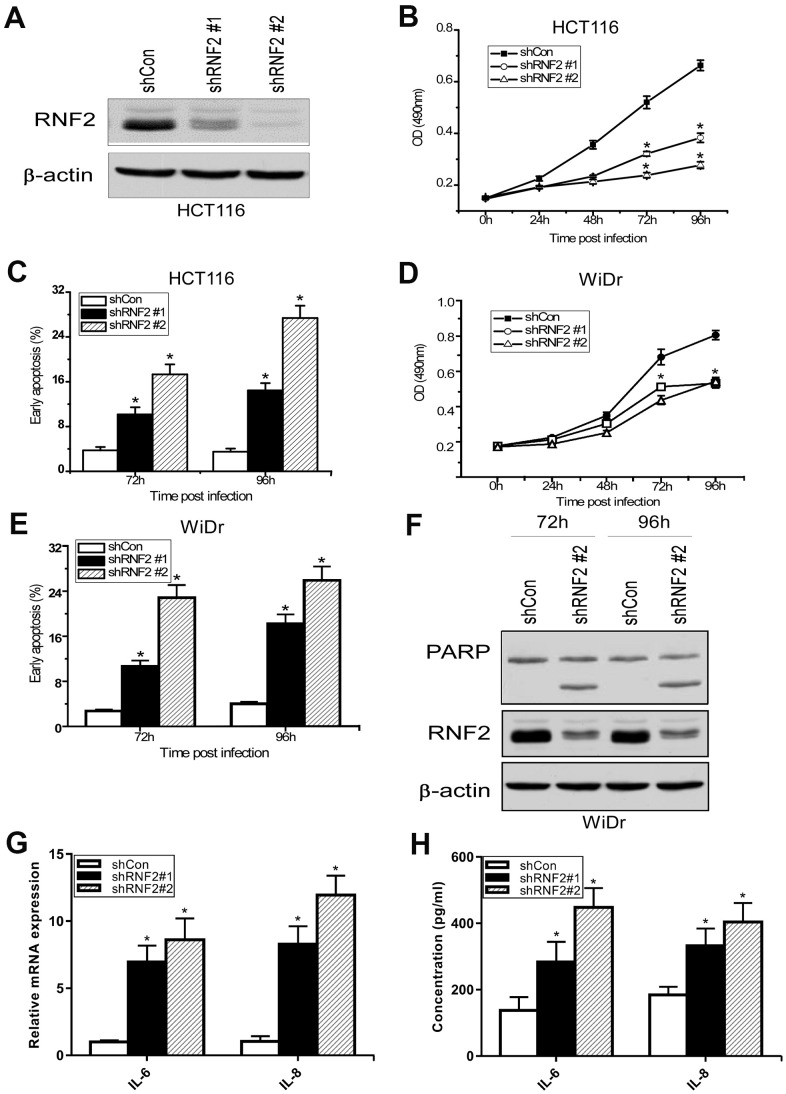
**The downregulation of RNF2 inhibited cell proliferation and increased apoptosis in CRC cells.** (**A**) Western blot analysis showing the knockdown efficiency of RNF2 in HCT116 cells that had been infected with shRNA-expressing lentiviruses for 48 hours. (**B**) MTT assay showing the proliferation of RNF2-knockdown and control HCT116 cells. (**C**) Apoptosis analysis of RNF2-knockdown and control HCT116 cells. (**D**) MTT assay showing the proliferation of RNF2-knockdown and control WiDr cells. (**E**) Apoptosis analysis of RNF2-knockdown and control WiDr cells. (**F**) Western blot analysis showing the knockdown efficiency of RNF2 and the cleavage of PARP in RNF2-knockdown and control WiDr cells. (**G**) qRT-PCR analysis showing the mRNA levels of IL-6 and IL-8 in RNF2-knockdown and control HCT116 cells. (**H**) ELISA assay showing the soluble IL-6 and IL-8 levels in culture media. Three independent experiments were performed and analyzed for B-E. Data represent the mean ± standard deviation. *p<0.05 versus shCon group.

To study the function of RNF2 in CRC cells, we first assessed RNF2 levels in several CRC cell lines and a normal colon cell line (CRL-1459). RNF2 levels were obviously higher in all the CRC cells we examined than in normal colon cells (Supplementary Figure 2A). Then, we used short hairpin RNA (shRNA)-expressing lentiviruses to knock down RNF2 in HCT116 and WiDr cells, and confirmed the knockdown efficiency using Western blotting (Figure 2A, 2F). A 3-(4,5-dimethylthiazol-2-yl)-2,5-diphenyltetrazolium bromide (MTT) assay demonstrated that cell proliferation was dramatically inhibited in RNF2-knockdown HCT116 and WiDr cells (Figure 2B, 2D). To evaluate the effects of RNF2 downregulation on the survival of cells, we used flow cytometry to analyze apoptosis. The results indicated that knocking down RNF2 increased apoptosis in both HCT116 and WiDr cells, based on the increased Annexin V^+^/propidium iodide^-^ cell percentages (Figure 2C, 2E). We also examined the cell cycle, and found that the proportion of cells in sub-G1 phase was greater in RNF2-knockdown cells than in control cells, further confirming that apoptosis was induced in RNF2-knockdown cells (Supplementary Figure 2B).

Next, we performed Western blotting, which revealed that both the mono-ubiquitination of H2A K119 and the expression of the cell cycle-related protein p21 were upregulated in RNF2-knockdown cells (Supplementary Figure 2C). We also used quantitative real-time PCR (qRT-PCR) and enzyme-linked immunosorbent assays (ELISAs) to analyze the mRNA and secreted protein levels of interleukin (IL)-6 and IL-8, which are markers of the senescence-associated secretory phenotype. Both the mRNA and secreted protein levels of IL-6 and IL-8 were upregulated in RNF2-knockdown HCT116 cells (Figure 2G, 2H). These results indicated that RNF2 promotes cell proliferation and inhibits apoptosis and senescence in CRC cells.

### The downregulation of RNF2 induced EGR1 expression

**Figure 3:**
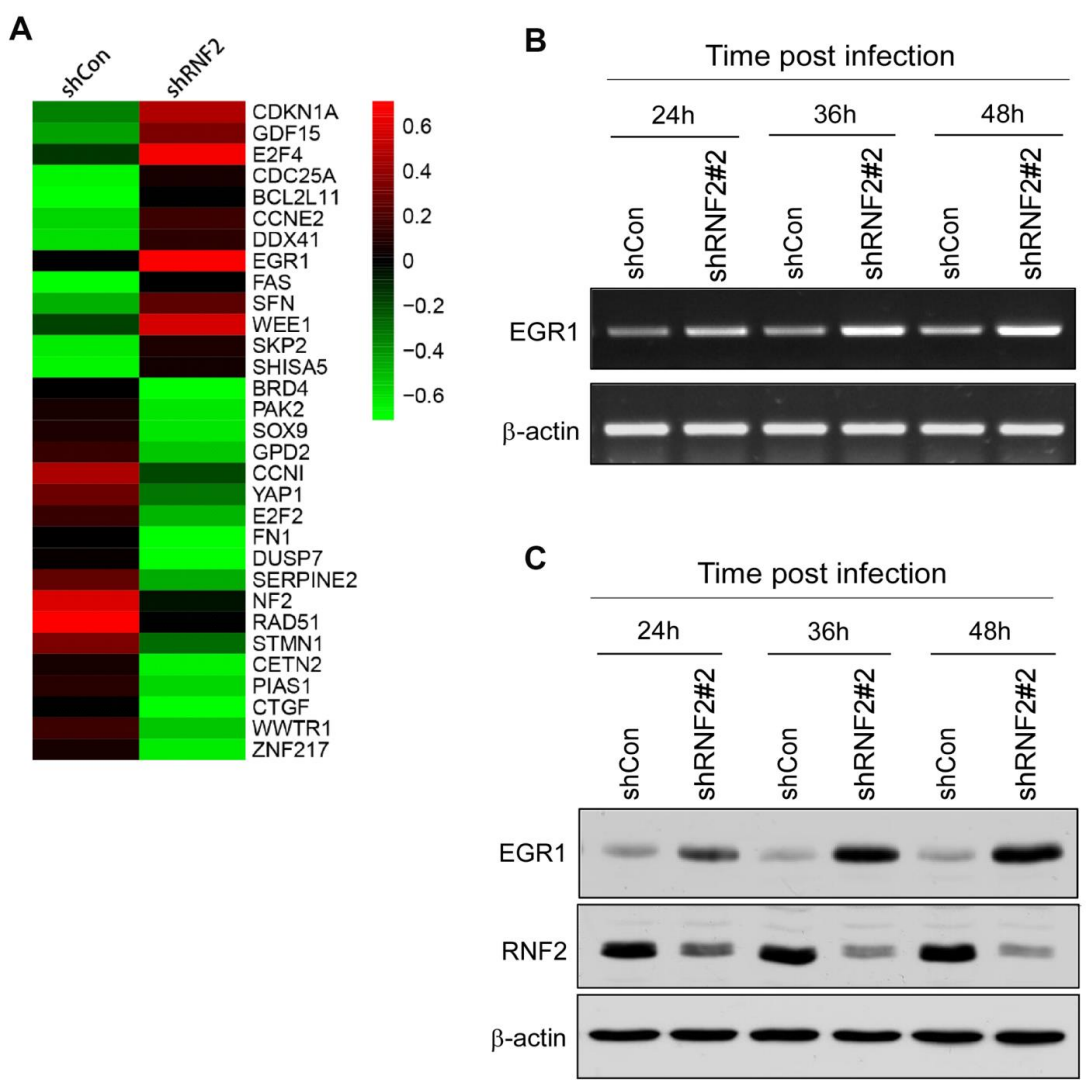
**The downregulation of RNF2 induced EGR1 expression.** (**A**) Heat map of some of the most differentially expressed genes in shRNF2-treated HCT116 cells, determined through microarray analyses (red: upregulated; green: downregulated). (**B**) RT-PCR analysis showing the increased EGR1 mRNA levels in RNF2-knockdown HCT116 cells. (**C**) Western blot showing the increased EGR1 protein levels in RNF2-knockdown HCT116 cells.

To investigate the molecular pathways through which RNF2 induced cell proliferation and suppressed apoptosis and senescence in CRC, we conducted a gene microarray analysis using mRNAs from RNF2-knockdown and control HCT116 cells. When a 1.5-fold difference in expression was used as the cutoff, 544 genes were differentially expressed between RNF2-knockdown cells and control cells, with 135 being upregulated and 409 being downregulated in RNF2-knockdown cells. These differentially expressed genes were evaluated using Gene Ontology and Kyoto Encyclopedia of Genes and Genomes analyses (Supplementary Figure 3A–3D). Some of the most significantly changed genes that are involved in cell proliferation and apoptosis are shown in Figure 3A.

Since PcG proteins transcriptionally repress gene expression, we concentrated on several tumor suppressors that were upregulated in RNF2-knockdown cells. EGR1 expression was significantly upregulated in RNF2-knockdown HCT116 and WiDr cells. In RNF2-knockdown HCT116 cells, the mRNA and protein levels of RNF2 decreased gradually over time after the lentiviral infection, while the mRNA and protein levels of EGR1 gradually increased (Figure 3B, 3C and Supplementary Figure 4A, 4B).

Then, we analyzed EGR1 expression and its correlation with RNF2 expression in CRC tissues. Among the 313 CRC tissues, 52.1% (163/313) were RNF2-positive, while 56.7% were EGR1-negative (178/313). A correlation analysis indicated that RNF2 expression correlated negatively with EGR1 expression in CRC tissues (r = -0.12; Supplementary Figure 5A, 5B). These results indicated that RNF2 may downregulate EGR1 in CRC.

### Knocking down EGR1 partially reversed the inhibition of cell proliferation and the increase in apoptosis in RNF2-knockdown cells

**Figure 4:**
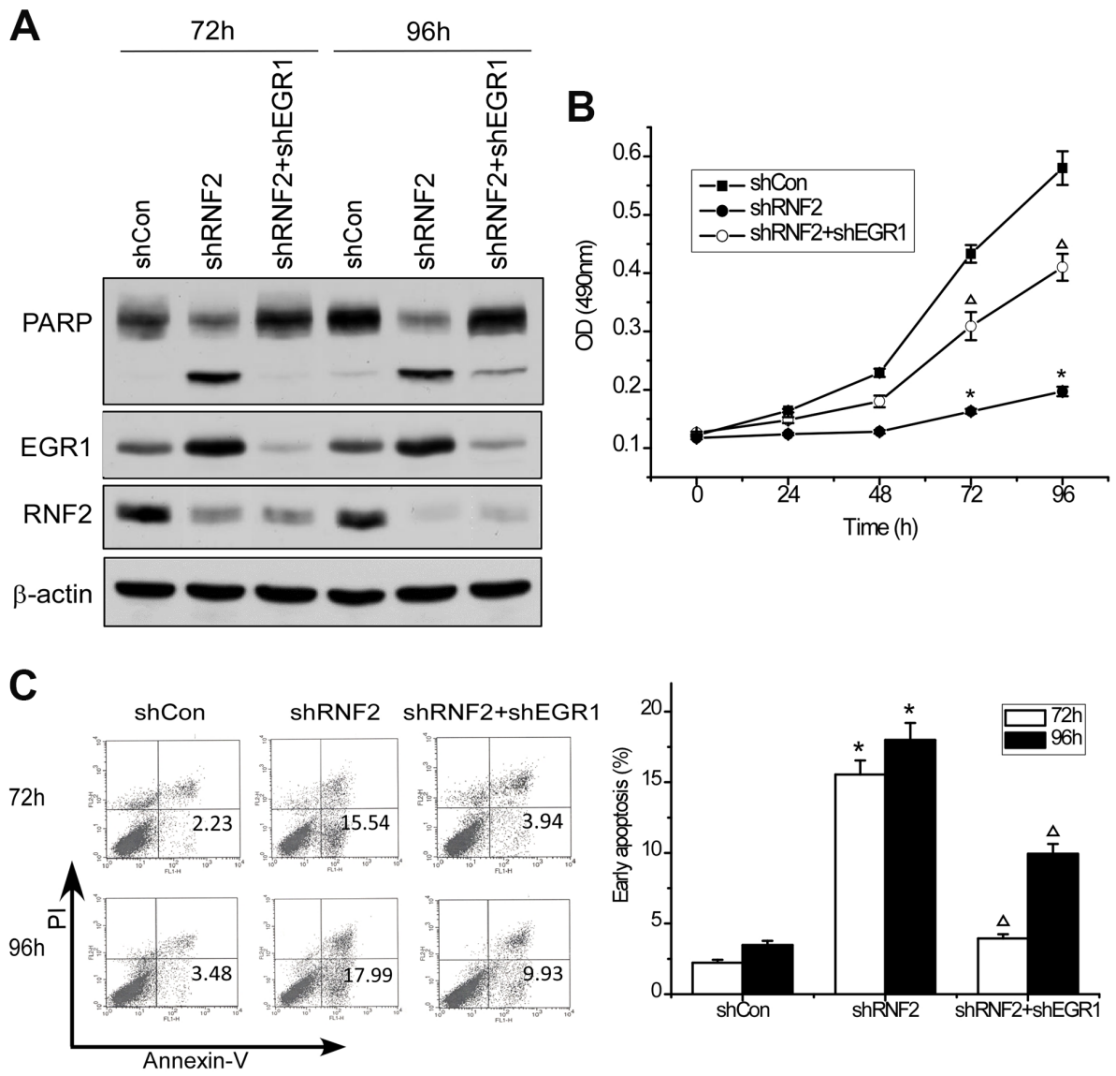
**Knocking down EGR1 partially reversed the inhibition of cell proliferation and the increase in apoptosis in RNF2-knockdown cells.** (**A**) Western blot showing the knockdown efficiency of RNF2 and EGR1 and the cleavage of PARP in RNF2/EGR1 double-knockdown, RNF2-knockdown and control HCT116 cells. (**B**) MTT assay showing the proliferation of RNF2/EGR1 double-knockdown, RNF2-knockdown and control HCT116 cells. (**C**) Apoptosis analysis of RNF2/EGR1 double-knockdown, RNF2-knockdown and control HCT116 cells. Three independent experiments were performed and analyzed for B and C. Data represent the mean ± standard deviation. *p<0.05 versus shCon, Δp<0.05 versus shRNF2.

To determine whether EGR1 is a downstream target of RNF2, we performed rescue experiments by simultaneously knocking down EGR1 and RNF2 in HCT116 cells. Western blotting confirmed the knockdown efficiency (Figure 4A). An MTT assay demonstrated that knocking down EGR1 partially reversed the inhibition of cell proliferation in RNF2-knockdown cells (Figure 4B). Flow cytometry indicated that knocking down EGR1 partially reversed the increase in apoptosis in RNF2-knockdown cells (Figure 4C). Poly(ADP-ribose) polymerase (PARP) cleavage was also reduced in the EGR1/RNF2 double-knockdown cells, further demonstrating the partial inhibition of apoptosis (Figure 4A). These findings demonstrated that RNF2 promotes cell proliferation and inhibits apoptosis by downregulating EGR1 in CRC cells.

### The downregulation of RNF2 reduced both RNF2 enrichment and H2A mono-ubiquitination at the EGR1 promoter

**Figure 5:**
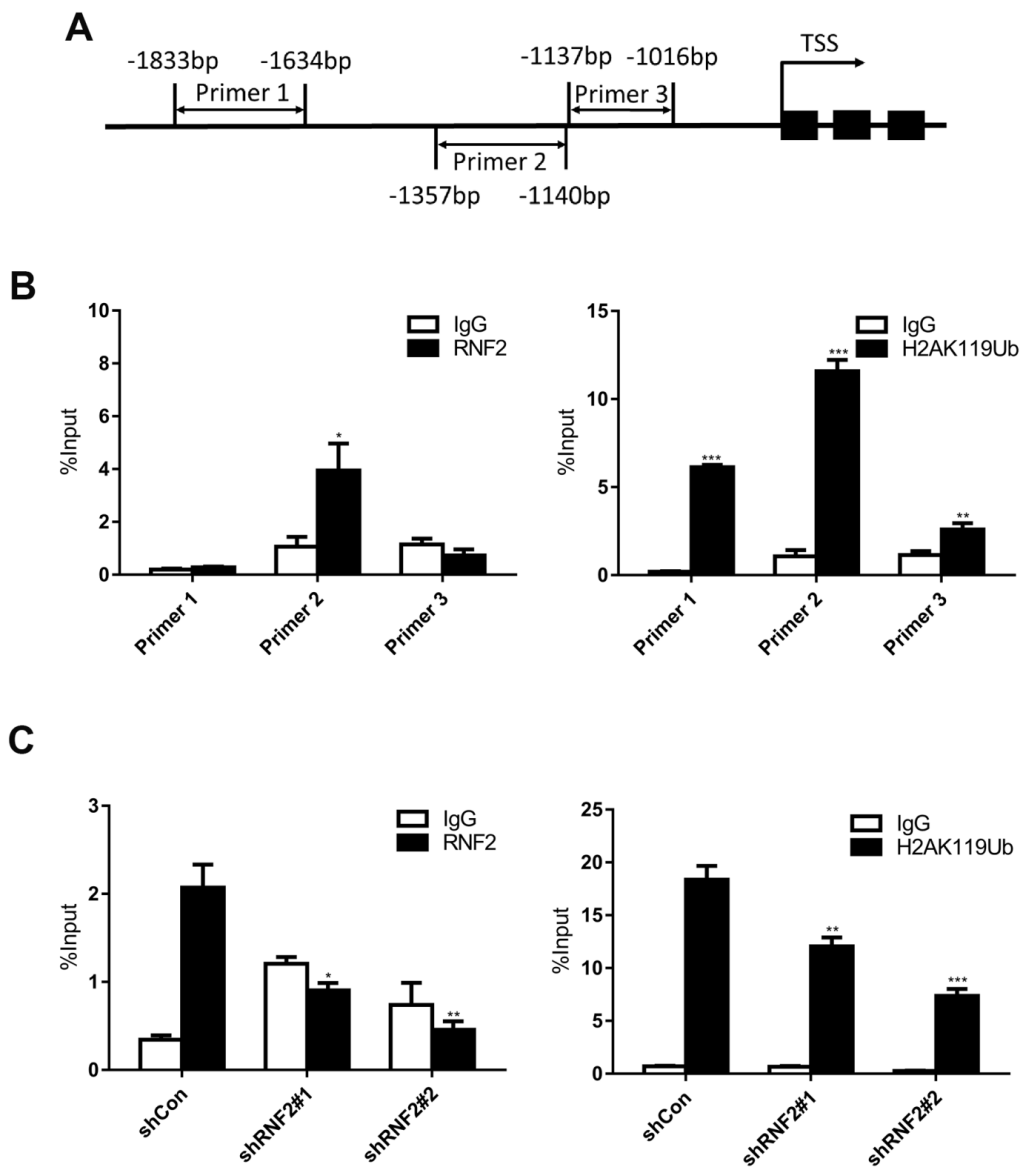
**The downregulation of RNF2 reduced both RNF2 enrichment and H2A mono-ubiquitination at the EGR1 promoter.** (**A**) The positions of the three PCR primer sets used in the ChIP assay. (**B**) Quantitative PCR analysis after a ChIP assay using antibodies against RNF2 and H2A K119Ub. Three independent experiments were performed and analyzed. Data represent the mean ± standard deviation. *p<0.05, **p<0.01 and ***p<0.001 versus IgG. (**C**) Quantitative PCR analysis using primer set 2 after a ChIP assay to show the enrichment of RNF2 and the mono-ubiquitination of H2A at the EGR1 promotor in RNF2-knockdown and control HCT116 cells. Three independent experiments were performed and analyzed. Data represent the mean ± standard deviation. *p<0.05, **p<0.01 and ***p<0.001 versus shCon.

To assess whether RNF2 directly inhibits EGR1 expression, we performed chromatin immunoprecipitation (ChIP) assays using antibodies against RNF2 and mono-ubiquitinated H2A K119 (H2A K119Ub). We designed three primer pairs to amplify specific regions up to 2000 base pairs upstream of the transcription start site of EGR1 (Figure 5A). We found that RNF2 and mono-ubiquitinated H2A were specifically enriched in the same region of the EGR1 promoter (Figure 5B). We also performed ChIP assays in control and RNF2-knockdown HCT116 cells, and observed that both RNF2 enrichment and H2A mono-ubiquitination at the EGR1 promoter were inhibited in RNF2-knockdown cells (Figure 5C). These findings demonstrated that RNF2 binds directly to the EGR1 promoter, where it mono-ubiquitinates H2A, thus inhibiting EGR1 expression.

## DISCUSSION

Epigenetic modifications such as DNA methylation, histone modification and small non-coding RNA regulation are all major contributors to tumor occurrence and progression [5, 6]. PcG proteins are important epigenetic regulators that repress transcription mainly by modifying histones, and several of these proteins are upregulated in and involved in the progression of numerous cancer types. Several studies have demonstrated that the expression of RNF2, a PcG protein, is associated with the tumor grade and prognosis, and could serve as a prognostic biomarker and therapeutic target in certain cancer types [22–24, 29, 30]. However, the function of RNF2 in CRC has not been determined.

We first evaluated RNF2 expression in 313 paired CRC tumor tissues and adjacent normal tissues, and observed that RNF2 was markedly upregulated in CRC tumor tissues. RNF2 expression was associated with clinicopathologic features such as the tumor differentiation status, TNM stage and Duke’s stage. In addition, RNF2 expression was associated with patients’ prognoses, as the survival times tended to be shorter in RNF2-positive patients than in RNF2-negative patients. We also studied RNF2 function by using shRNA to knock down RNF2 in CRC cells, and observed that the downregulation of RNF2 inhibited cell proliferation, promoted apoptosis and induced senescence in CRC cells.

We then performed a gene microarray analysis to identify the molecular pathways through which RNF2 altered CRC cell proliferation, apoptosis and senescence. In total, 554 genes exhibited an expression difference of at least 1.5-fold between RNF2-knockdown cells and control cells. Considering that PcG proteins repress gene transcription, we mostly concentrated on tumor suppressors that were upregulated in RNF2-knockdown cells. EGR1 expression was clearly upregulated in RNF2-knockdown HCT116 and WiDr cells; thus, we further explored whether EGR1 was a target of RNF2.

EGR1 is a transcription factor in the immediate-early gene group, and alters the expression of various genes involved in development, cellular differentiation and proliferation [31–33]. EGR1-knockout mice exhibit accelerated tumor development, and p53 has been identified as a direct target of EGR1 [34, 35]. Several other tumor suppressors are also targets of EGR1, including transforming growth factor-β, phosphatase and tensin homolog, p73 and fibronectin [36]. EGR1, p53 and p73 have been found to network to induce apoptosis in tumor cells [37]. EGR1 also binds to the promoter of CDKN2B and the first intron of CDKN1A to upregulate their expression and stimulate RAF-induced senescence [38].

Because it upregulates several tumor suppressor genes, EGR1 is also considered to be a tumor suppressor, and lower EGR1 expression has been associated with poorer outcomes in many cancer types, including non-small-cell lung cancer, osteosarcoma, glioma and breast cancer [39–42]. However, some studies have indicated that EGR1 may accelerate tumor progression by stimulating cellular proliferation, invasion and angiogenesis in certain cancer types, such as prostate, ovarian, gastric and liver cancers [43–47]. In CRC, EGR1 has been found to promote or suppress tumor growth, depending on the cell type and environment. For example, Kim et al. reported that EGR1 overexpression promoted the growth of LS174T cells, indicating that EGR1 has an oncogenic function [48]. However, Lee et al. demonstrated that EGR1 overexpression stimulated apoptosis, while EGR1 silencing prevented apoptosis in tolfenamic acid-treated CRC cells [49]. Han et al. found that sanguinarine markedly induced EGR1 expression, while knocking down EGR1 significantly inhibited sanguinarine-induced apoptosis in HCT116 cells [50]. In addition, Choi et al. found that 26.6% of CRC patients carried EGR1 frameshift mutations and mutational intratumoral heterogeneities [51]. Thus, several studies have indicated that EGR1 is a tumor suppressor in CRC.

In the present study, we found that EGR1 mRNA and protein levels were elevated in RNF2-knockdown CRC cells. Knocking down EGR1 partially reversed the inhibition of proliferation and the increase in apoptosis in RNF2-knockdown cells, confirming that EGR1 is a downstream target of RNF2. We also examined RNF2 and EGR1 levels in clinical CRC tissues, and detected a negative correlation between them. We then used ChIP assays to determine the mechanism whereby RNF2 suppressed EGR1 expression, and found that RNF2 was enriched at the EGR1 promoter. In RNF2-knockdown cells, both RNF2 enrichment and H2A mono-ubiquitination at the EGR1 promoter decreased, indicating that RNF2 downregulates EGR1 by mono-ubiquitinating H2A.

However, knocking down EGR1 did not completely reverse the inhibition of cell proliferation and the increase in apoptosis in RNF2-knockdown cells, indicating that RNF2 may also function through other mechanisms. For example, p21 was also upregulated in RNF2-knockdown HCT116 cells, suggesting that p21 may be a downstream target of RNF2 in CRC, consistent with a previous study in hepatic stem/progenitor cells [27]. Since EGR1 has been demonstrated to upregulate CDKN1A [38], the increase in p21 expression may have been induced by the increase in EGR1 expression in RNF2-knockdown cells. Our gene microarray analysis also revealed other genes that may be targets of RNF2, so these genes deserve further evaluation.

EGR1 expression is regulated by several transcription factors, including ETS and activating transcription factor 3 [49, 52]. EGR1 expression can also be altered by different epigenetic modifications, such as histone acetylation, methylation and ubiquitination [53–55]. Further evaluation is needed to determine whether there is a correlation between transcription factor-induced regulation and epigenetic regulation. Recently, a PRC1-specific inhibitor, PRT4165 (2-pyridine-3-yl-methylene-indan-1,3-dione), was found to suppress RNF2-induced H2A ubiquitination, thus inhibiting DNA repair following DNA damage [56]. Considering our finding that downregulating RNF2 inhibited cell growth and induced apoptosis in CRC cells, further investigation is warranted to assess whether PRT4165 can inhibit cell growth, induce apoptosis and be applied in the treatment of CRC.

In summary, our study revealed that RNF2 expression was greater in CRC tumor tissues than in adjacent normal tissues, and was associated with the clinicopathologic features and prognoses of CRC patients. We demonstrated that RNF2 may exert its oncogenic functions by transcriptionally repressing EGR1 in CRC. Thus, RNF2 could be used as a novel prognostic biomarker and therapeutic target in CRC.

## MATERIALS AND METHODS

### Patients and follow-up

This study was approved by the ethics committee of the Fourth Military Medical University. All the involved patients gave their written informed consent. The CRC tissue microarrays contained 313 CRC tumor tissues and paired adjacent normal tissues that were collected from patients at the Department of Anorectal Surgery, Tianjin Union Medical Center, from February 2004 to December 2005. None of the patients were treated with chemotherapy or radiotherapy before surgery, and all of them received chemotherapy after surgery. The criteria of the Union for International Cancer Control were used to classify the histology and clinical stage of each patient. Each participant’s follow-up information was updated every four months through phone calls. The overall survival time was defined from the date of surgery to death. The deaths of patients were confirmed by their families.

### Immunohistochemistry

Immunohistochemistry was used to examine RNF2 expression in paraffin-embedded tissue microarray sections containing 313 CRC tumor tissues and adjacent normal colon tissues. In brief, the slides were deparaffinized in xylene and rehydrated using a graded alcohol series. Then, 3% H_2_O_2_ was used to block endogenous peroxidase activity, and pre-immune rabbit serum was used to block non-specific protein binding. The slides were then incubated with an anti-RNF2 antibody (Abcam, ab101273) overnight in a humidified chamber at 4° C. Subsequently, the slides were washed with phosphate-buffered saline (PBS) and incubated with a horseradish peroxidase-conjugated secondary antibody for 30 minutes at room temperature. Then, the slides were incubated with 3, 3'-diaminobenzidine chromogen for 2-3 minutes for visualization. The slides were counterstained with hematoxylin, and RNF2 expression was evaluated using the H-score method by two pathologists who were blinded to the patients’ clinical information [19]. The H-score was calculated as the sum of the cells with strong signals (3×), moderate signals (2×) and weak signals (1×) in one hundred cells. The H-scores ranged from 0 to 300, and patients with H-scores higher than 50 were designated as RNF2-positive, while those with H-scores lower than 50 were designated as RNF2-negative.

### Cell culture

Human colon cancer cells (HCT116 and WiDr) were obtained from the Type Culture Collection of the Chinese Academy of Sciences (Shanghai, China). The cells were cultured in McCoy’s 5A medium (HCT116) or Eagle’s minimum essential medium (WiDr) supplemented with 10% fetal bovine serum and 1% penicillin-streptomycin in a humidified incubator containing 5% CO_2_ at 37° C.

### Lentiviral packaging and cell infection

Lentiviral plasmids expressing shRNA against RNF2 (shRNF2 #1, #2) were purchased from Open Biosystems (Huntsville, AL, USA). Lipofectamine 2000 (Invitrogen, Carlsbad, CA, USA) or JetPEI (Polyplus Transfection, New York, NY, USA) was used to transfect cells with the plasmid DNA. For virus packaging, the shRNF2-containing lentiviral plasmids were co-transfected with packaging and envelope plasmids (psPAX.2 and pMD2.G) into 293T cells. The lentiviruses were collected 36 and 60 hours after transfection, and were centrifuged at 1000 rpm for 5 minutes before the supernatants were harvested. HCT116 and WiDr cells were infected with the lentiviruses, together with 8 μg/mL polybrene (Millipore, Billerica, MA, USA).

### RT-PCR

Total RNA was extracted with an RNeasy Plus Universal Mini Kit (QIAGEN, Hilden, Germany). The quantity and quality of the RNA were examined with a NanoDrop 2000 (Thermo Scientific, USA). The RNA was reverse-transcribed into cDNA using a Revert Aid^TM^ First Strand cDNA Synthesis Kit (Fermentas, St. Leon-Rot, Germany), and used as a template. The PCR primer sequences were as follows: EGR1, Forward: 5’-CTGACCGCAGAGTCTTTTCCTG-3’, Reverse: 5’-CTGACCGCAGAGTCTTTTCCTG-3’; β-actin, Forward: 5’-CCGTGTGAACCATGTGACTT-3’, Reverse: 5’-CTAAGTTGCCA GCCCTCCTA-3’.

### Western blot

Radioimmunoprecipitation assay lysis buffer supplemented with a protease inhibitor cocktail (Roche, Indianapolis, IN, USA) was used for cell lysate extraction. The protein concentration was determined using a bicinchoninic acid assay. The proteins from the cell lysates were electrophoretically separated on 10% sodium dodecyl sulfate polyacrylamide gels and transferred onto nitrocellulose membranes (Millipore, Bedford, MA, USA). The membranes were blocked in 5% nonfat milk for 1 hour at room temperature before they were incubated with primary antibodies overnight at 4° C. The antibodies used in this study included anti-RNF2 antibody (CST, #5694), anti-EGR1 antibody (CST, #4153) and anti-β-actin antibody (Sigma, A5441). The membranes were then washed three times with Tris-buffered saline-Tween and incubated with a horseradish peroxidase-conjugated secondary antibody for 1 hour at room temperature. Protein bands were visualized and photographed using a FluorChem FC2 system (Alpha Innotech, San Leandro, CA, USA).

### MTT assay

An MTT assay was used to assess *in vitro* cell proliferation. Cells that had been infected with lentiviruses (control [shCon], shRNF2 and/or shEGR1) for 24 hours were plated on 96-well plates at a density of 3×10^4^ cells/mL, 100 μL per well. At the timepoint of examination, each well was treated with 20 μL of MTT substrate (from a 2.5 mg/mL stock solution in PBS), and the plates were placed in an incubator for 4 hours. Then, the culture medium was replaced with 150 μL of dimethylsulfoxide, and the plates were gently shaken for 15 minutes before the absorbance at 492 nm was obtained using a spectrophotometer. The plates were analyzed at the indicated timepoints for five consecutive days.

### Apoptosis and cell cycle analysis

HCT116 or WiDr cells were infected with shRNA lentiviruses (shCon, shRNF2 and/or shEGR1) for 72 or 96 hours before analysis. For the analysis of apoptosis, the cells were trypsinized, counted and suspended in PBS (1×10^6^ cells per group). Then, the cells were incubated with Annexin V-fluorescein isothiocyanate and propidium iodide (BD Biosciences, CA, USA) for 15 minutes in the dark at room temperature. Apoptotic cells were then assessed on a flow cytometer (CYTOMICS FC 500, Beckman Coulter). For the analysis of the cell cycle, cells were harvested, washed with ice-cold PBS and fixed with 70% ice-cold ethanol. Then, the cells were centrifuged, resuspended in PBS containing RNase (100 μg/mL) and propidium iodide (40 μg/mL), and incubated at 37° C for 1 hour. The cell cycle was then analyzed using flow cytometry.

### ELISA

ELISA assays were used to evaluate the secreted protein levels of IL-6 and IL-8 in culture media from control and RNF2-knockdown cells. ELISA kits for human IL-6 and IL-8 were used in accordance with the manufacturer’s manual (CUSABIO BIOTEC Co., Ltd, Wuhan, China).

### Gene microarray analysis

Total RNA was extracted from HCT116 cells that had been infected with shCon or shRNF2 #2 for 48 hours. The RNA was then quantified and sent to Phalanx Biotech Group for gene expression analysis using a Human Whole Genome OneArray^TM^ (HOAv4.3, Phalanx Biotech Group, Taiwan). The RNA was amplified and hybridized with 10 μg of fragmented biotin-labeled complementary RNA at 50° C for 14-16 hours in triplicate. Then, non-specific binding targets were removed, and the hybridization arrays were conjugated with a Streptavidin-Cy3-labeled detector. The arrays were then dried and scanned on a DNA Microarray Scanner, and the data were quantified and analyzed.

### ChIP assay

The ChIP assay was conducted according to a previously published method, with slight modifications [28]. Briefly, HCT116 cells in the different groups were treated with 1% formaldehyde for 15 minutes at room temperature to cross-link proteins and DNA. The cells were then harvested, centrifuged and resuspended in radioimmunoprecipitation assay buffer containing a protease inhibitor cocktail. The cell lysates were sonicated to ensure that the chromatin was sheared to a length of 200-1000 base pairs on average. The sheared chromatin was then subjected to immunoprecipitation with different antibodies, including an anti-RNF2 antibody (CST, #5694), anti-H2A K119Ub antibody (CST, #8240) and control IgG (isotype control) (CST, #2729) with magnetic beads. The immunoprecipitants were eluted and reverse cross-linked, and the proteins were digested with proteinase K. The purified DNA was then used for RT-PCR. The primers used in this study were designed according to the sequence upstream of the transcription start site of EGR1. The primers were: Primer set 1, Forward: 5’-GGACAGCCACAGAGGGATTA-3’, Reverse: 5’-TCCAGAGGAGGTGCTGTTTT-3’; Primer set 2, Forward: 5’-CTGCTCAGTTCGTGCTCACT-3’, Reverse: 5’-GCTTCCCTATGGGCTGTCTG-3’; Primer set 3, Forward: 5’-CTCTTTCGGATTCCCGCAGT-3’, Reverse: 5’-CCCCAAGAGAGGCCTGATTC-3’.

### Statistical analysis

Statistical analyses were performed using IBM SPSS statistical software (version 20.0). Student’s t test was used for data analysis. Survival curves were generated using the Kaplan-Meier method, and distributions were compared using the log-rank test. The hazard ratios for factors associated with survival were determined using Cox proportional hazard models. Differences between two groups were analyzed using χ2 tests and Fisher’s exact tests. Correlations were assessed with Spearman’s correlation analysis. P values < 0.05 were considered to be statistically significant.

## Supplementary Material

Supplementary Figures
