# Supplementary material for: Ring finger protein 2 promotes colorectal cancer progression by suppressing early growth response 1
Source: Aging (Albany NY). 2020 Dec 19;12(24):26199–220. doi: 10.18632/aging.202396 (PMC7803491; doi:10.18632/aging.202396)
Supplement: Supplementary Figures [file aging-12-202396-s001.pdf]

SUPPLEMENTARY FIGURES

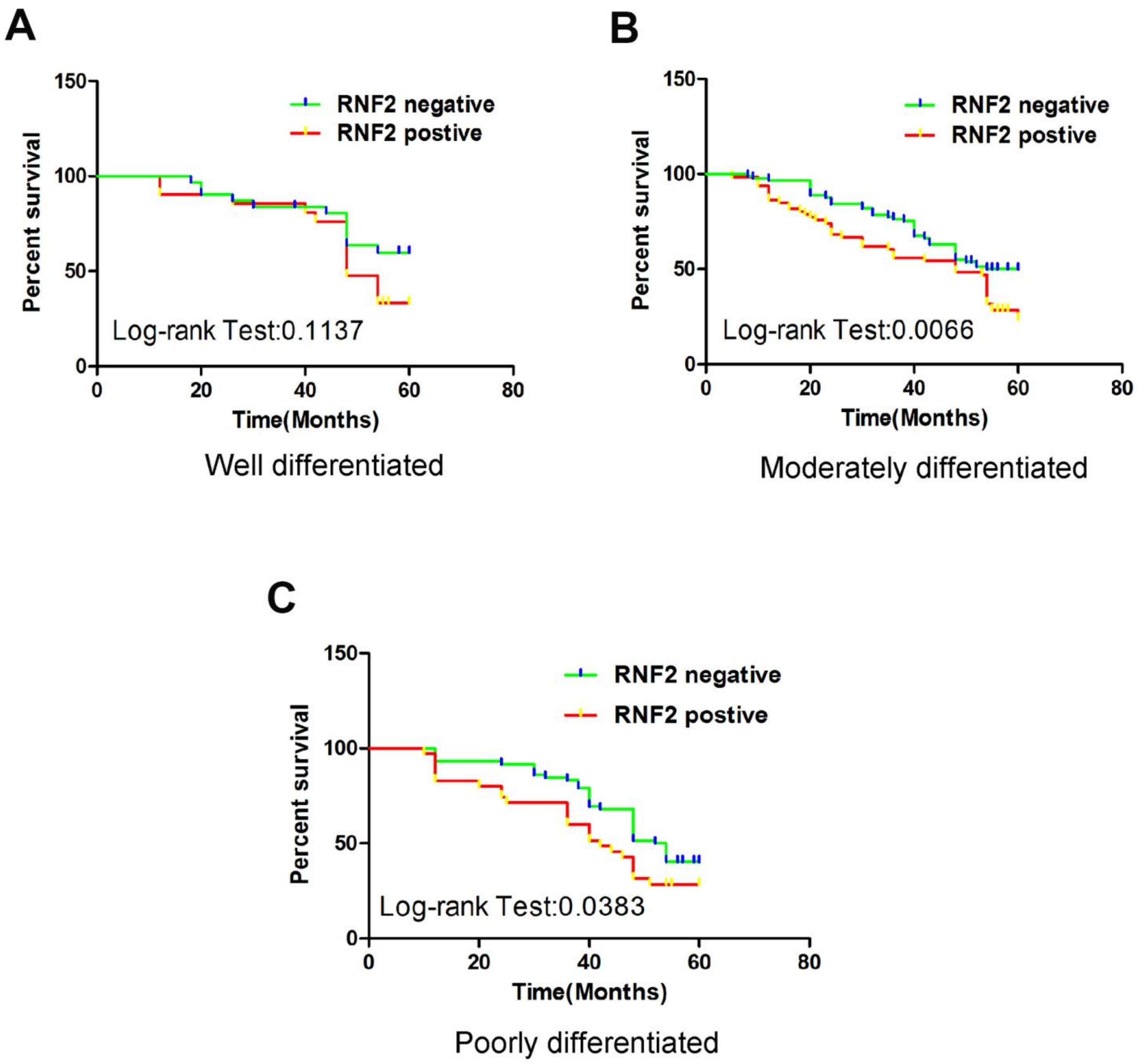

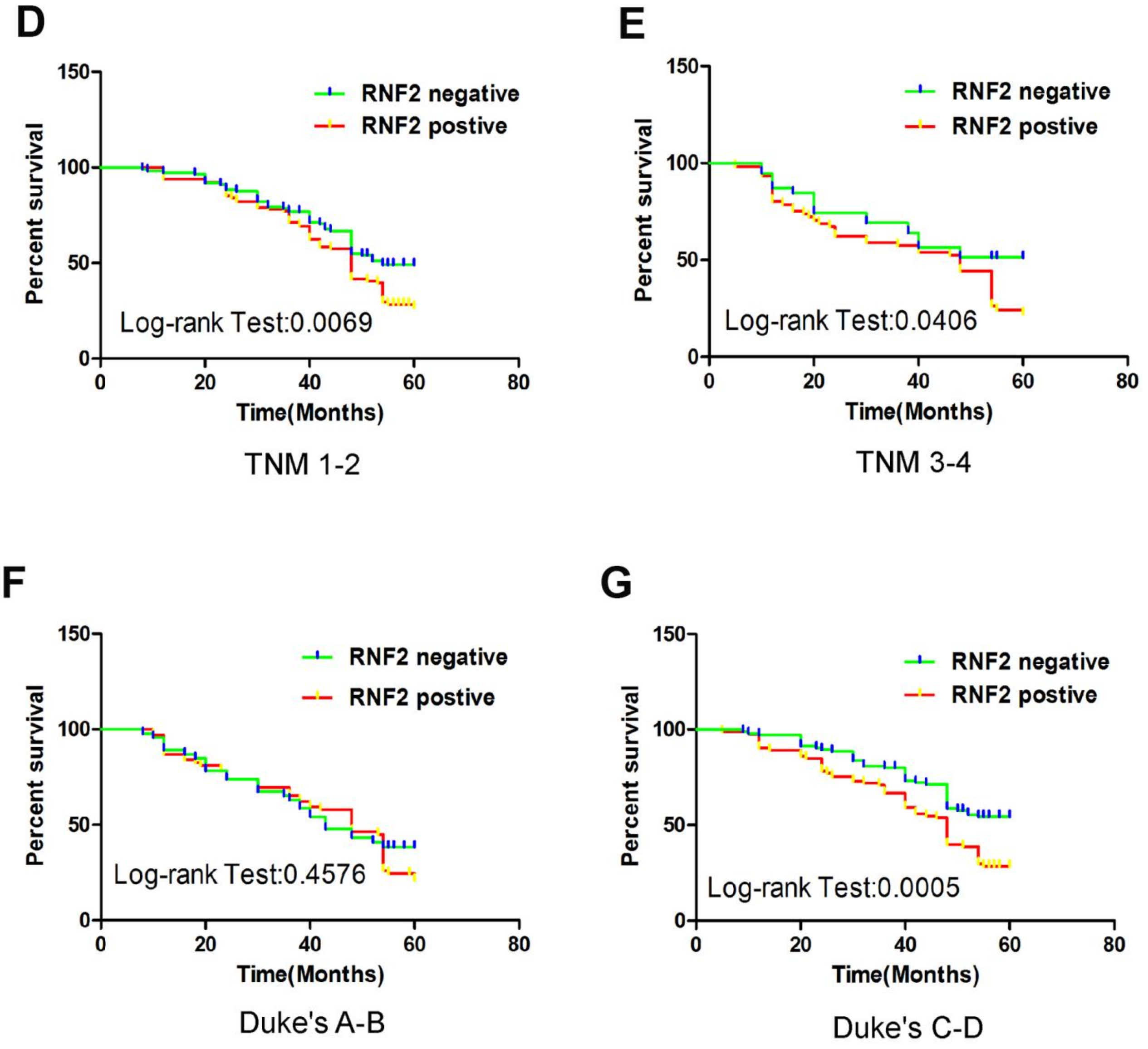

Supplementary Figure 1. The correlation between RNF2 expression and survival in patients with different differentiation status, TNM stage or Duke's stage. Patients' survival was calculated and plotted using Kaplan-Meier method. Patients were determined as RNF2 positive or RNF2 negative based on RNF2 IHC staining result. (**A**–**C**) Survival of patients with different differentiation status. In patients with moderately differentiated and poorly differentiated tumors, those had positive RNF2 expression showed worse survival. (**D**, **E**) Survival of patients in different TNM stage. In patients with tumors either in TNM 1-2 or TNM 3-4 stage, those had positive RNF2 expression showed worse survival. (**F**, **G**) Survival of patients in different Duke's stage. In patients with tumors in Duke's (**C**, **D**) stage, those had positive RNF2 expression showed worse survival.

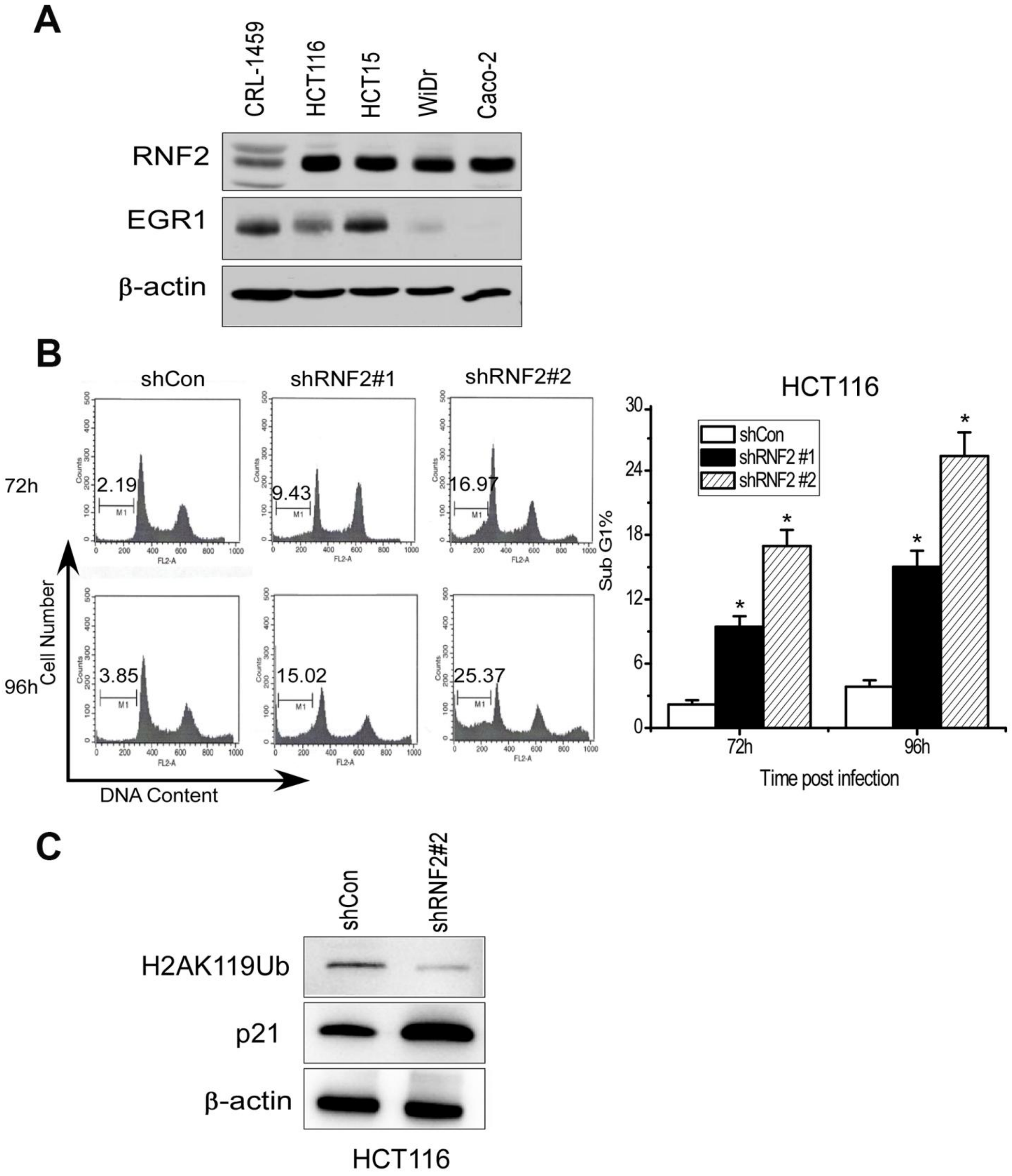

Supplementary Figure 2. Expression of RNF2 and EGR1 in CRC cells, and knockdown of RNF2 induced apoptosis in HCT116 cells. (**A**) Western blot analysis to show the expression of RNF2 and EGR1 in several CRC cell lines and normal colon cell CRL-1459. (**B**) Cell cycle analysis of control and RNF2 knockdown HCT116 cells. Data are shown as the mean±S.D. from three independent experiments. *p<0.05 versus shCon. (**C**) Western blot analysis to show the expression of p21 and H2AK119Ub in control and RNF2 knockdown HCT116 cells.

A GO enrichment analysis of the differentially expressed genes (Cluster 1)

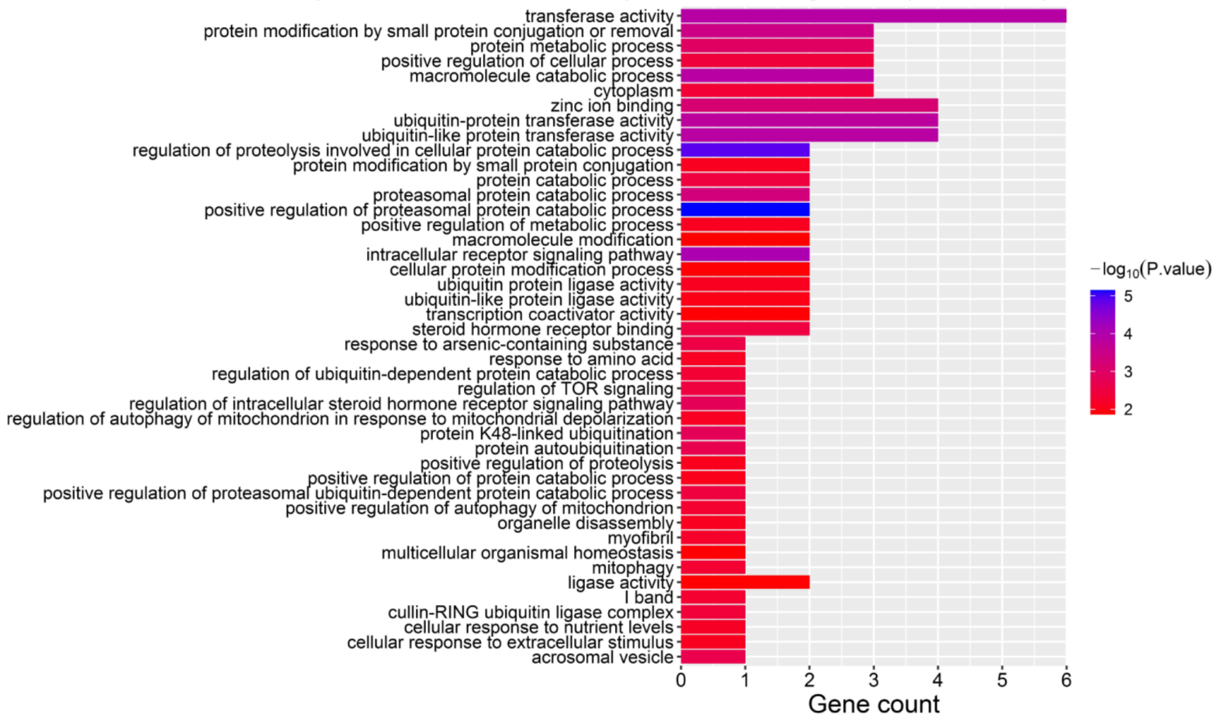

B GO enrichment analysis of the differentially expressed genes (Cluster 2)

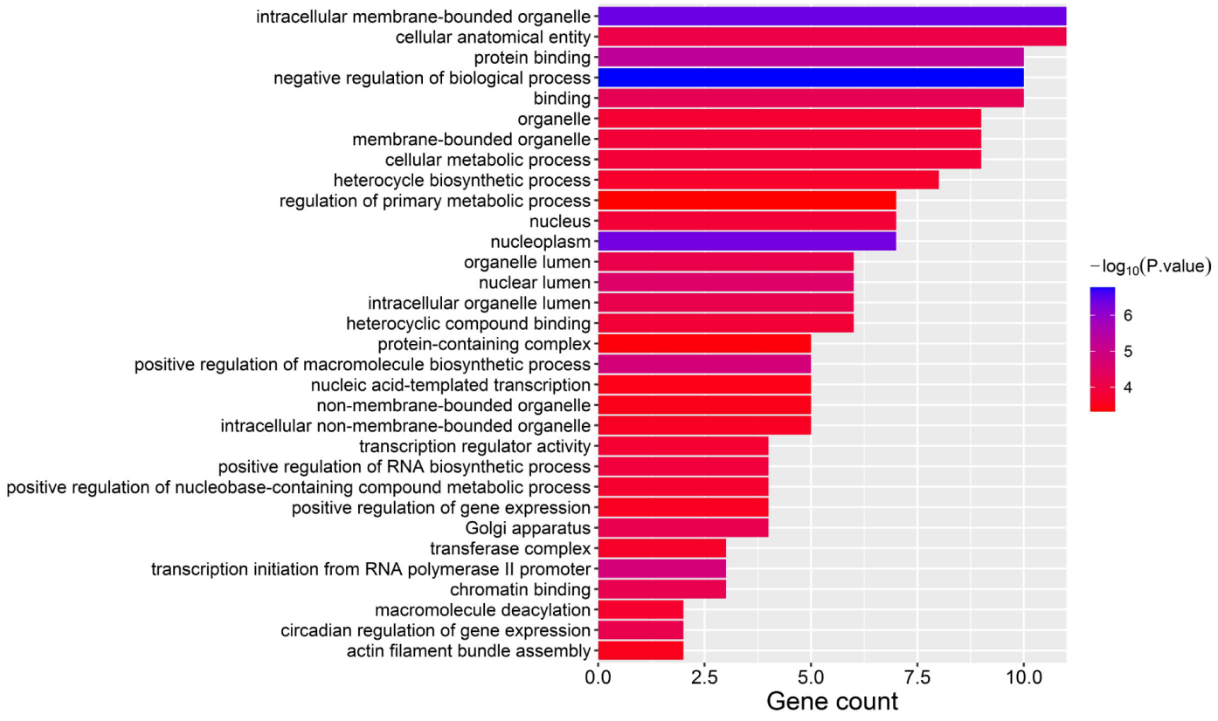

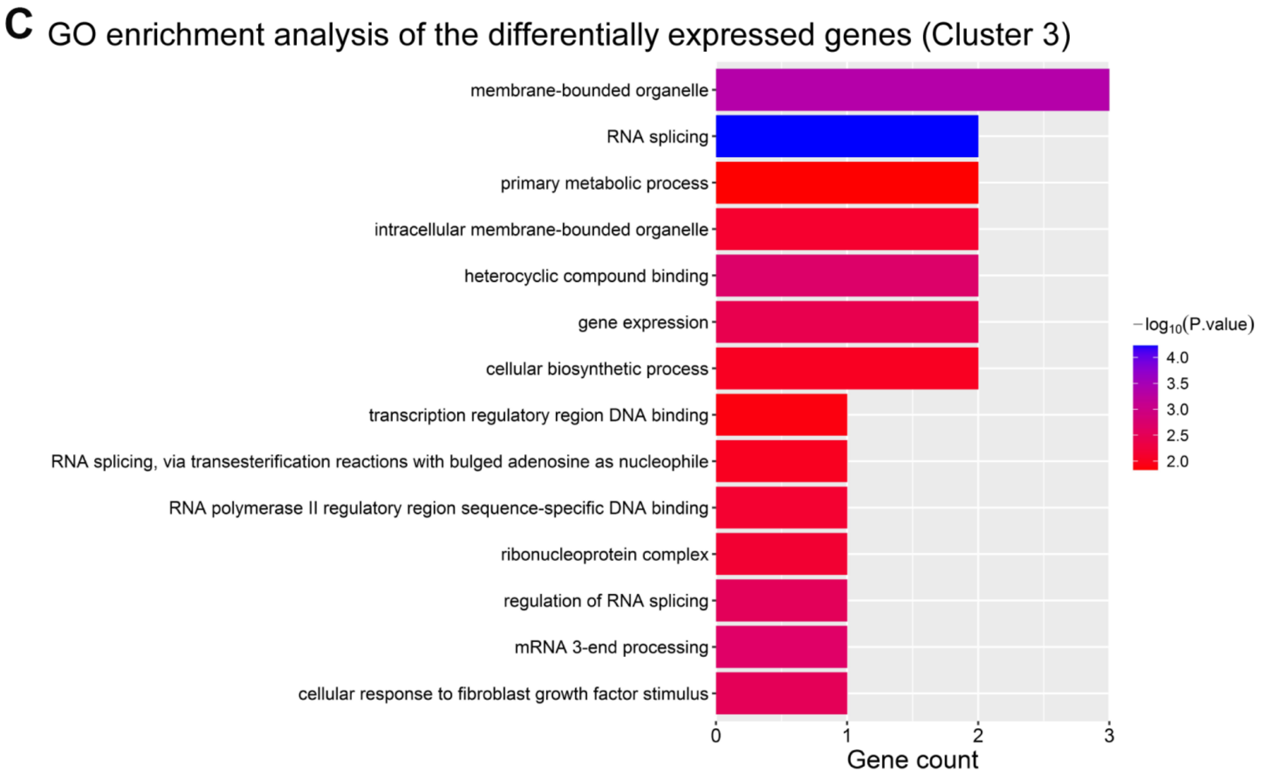

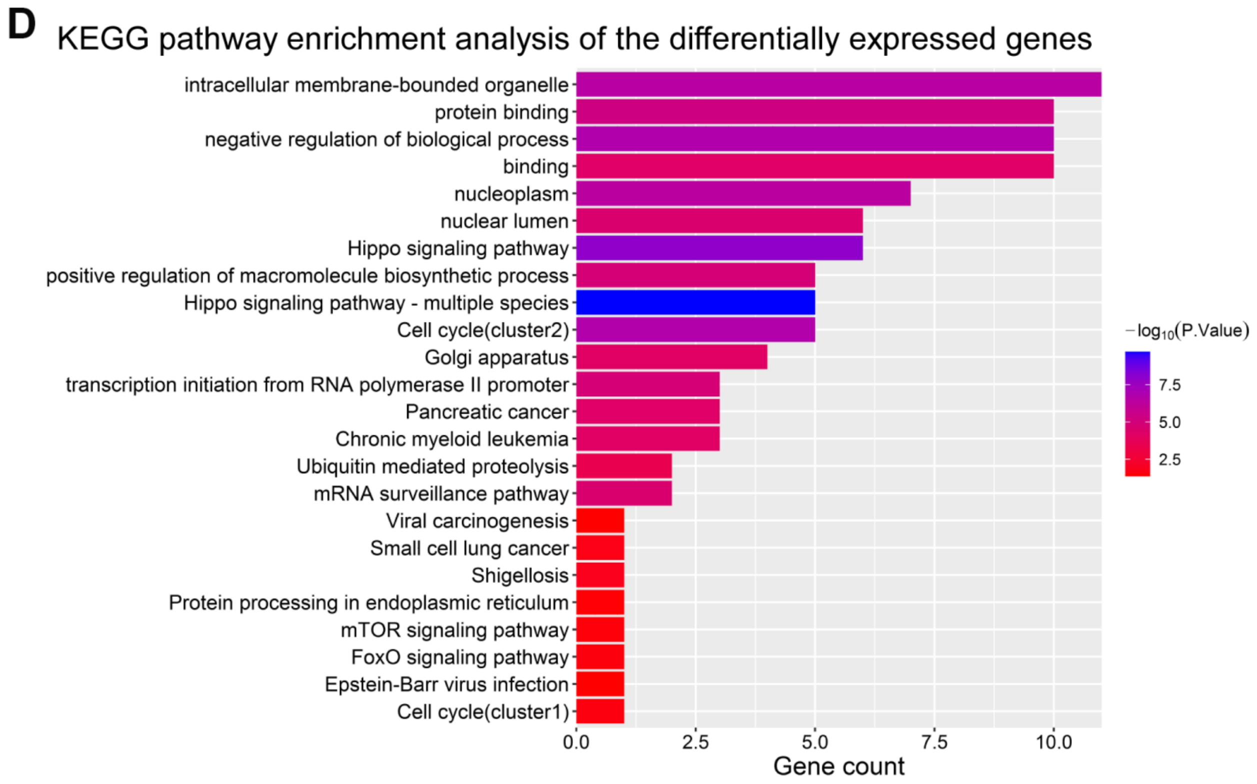

Supplementary Figure 3. GO biological process and KEGG pathway enrichment for the differentially expressed genes. (**A**–**C**) GO enrichment analysis of the differentially expressed genes in RNF2 knockdown cells (Cluster 1-3). The x axis indicates the number of genes within each GO term. (**D**) KEGG pathway enrichment analysis of the differentially expressed genes in RNF2 knockdown cells. The x axis indicates the number of genes within each KEGG term.

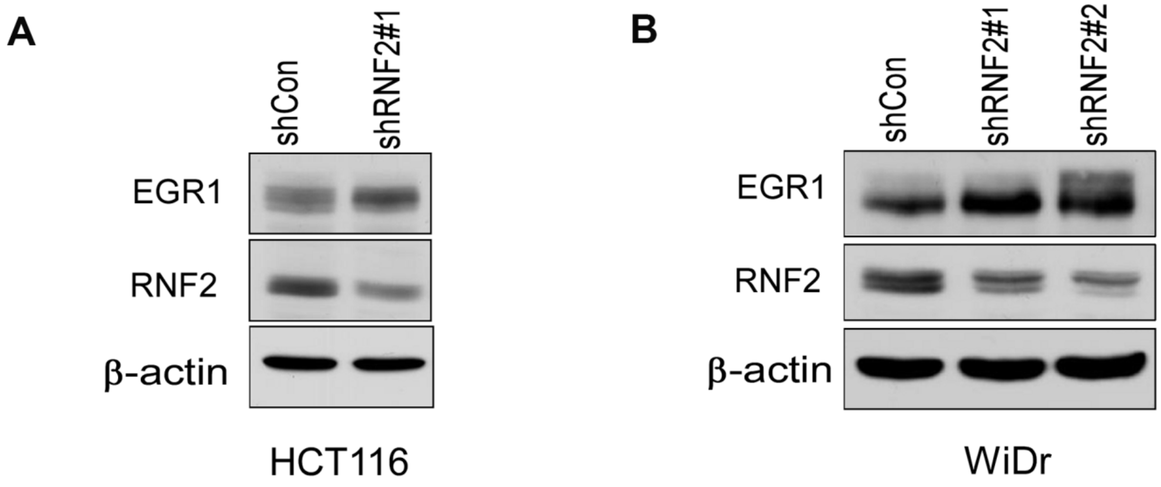

Supplementary Figure 4. Downregulation of RNF2 resulted in upregulation of EGR1 in both HCT116 and WiDr cells. (**A**) Western blot to show the increased EGR1 expression in RNF2 knockdown HCT116 cells (by shRNF2 #1). (**B**) Western blot to show the increased EGR1 expression in RNF2 knockdown WiDr cells.

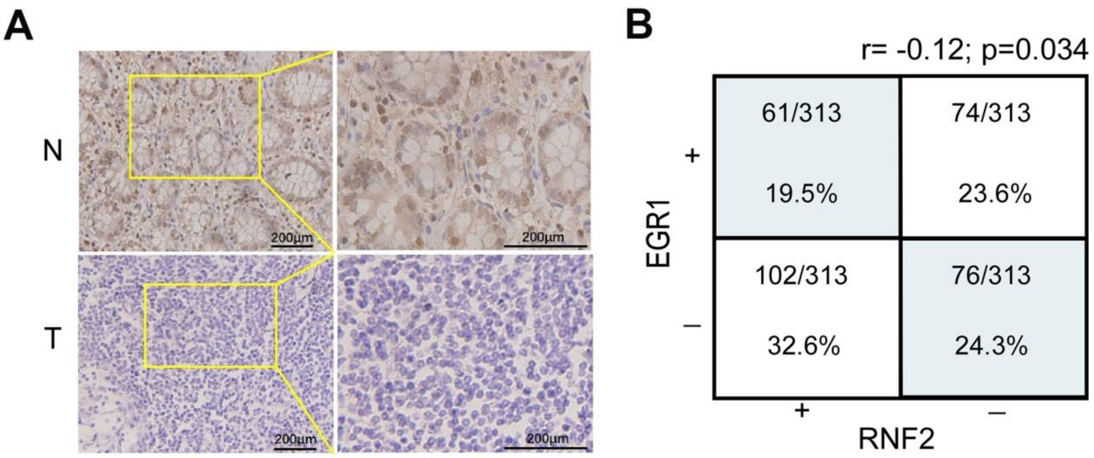

Supplementary Figure 5. Correlation analysis of RNF2 and EGR1 expression in clinical CRC tissues. (**A**) Representative IHC staining results to show negative and positive EGR1 expression in clinical CRC tumor tissues and adjacent normal tissues. Right pictures are the magnified view of the yellow box in the left. Scale bar: 200 μm. (**B**) Correlation analysis of RNF2 and EGR1 expression in clinical CRC tissues with r = -0.12 and p= 0.034.
